# Migration and Adaptation as Indicators of Social Mobility Migrants

**DOI:** 10.3390/bs10010030

**Published:** 2020-01-09

**Authors:** Svetlana Gurieva, Kristi Kõiv, Olga Tararukhina

**Affiliations:** 1 Phycology Division, Saint Petersburg State University, Universitetskaiya nab. 7/9, 199034 Saint Petersburg, Russia; 2 Institute of Education, University of Tartu, 50090 Tartu, Estonia; kristi.koiv@ut.ee; 3 Saint Petersburg State University, Universitetskaiya nab. 7/9, 199034 Saint Petersburg, Russia; tararukhina@gmail.com

**Keywords:** migration processes, social mobility, labor migrants, adaptation

## Abstract

The economic and social changes in modern society have resulted in intensive and extensive migrant activity. The article contains a review of social, psychological, and gender aspects of migration from three countries of Central Asia (former Soviet republic)—Kyrgyzstan, Tajikistan, and Uzbekistan—in Russia (St. Petersburg). The main objective of our study was to identify socio-psychological mechanisms of migration from Central Asia—the general and specific peculiarities of the acculturation process of migrant workers. Participants in the study were labor migrants from Tajikistan, Kyrgyzstan, and Uzbekistan. The research was conducted in St. Petersburg. In total, 98 people aged from 19 to 42 years old took part in the research (median age = 32.26, SD = 3.44), among them, women made up 44% and men made up 56%. Three ethnic groups were represented in the sample: Kyrgyz people (34 persons), Tajik people (32 persons), and Uzbek people (32 persons). The research found both general and specific features related to certain ethnic groups. The research results showed that there were significant differences between the migrants from Kyrgyzstan, Tajikistan, and Uzbekistan by the following acculturation indicators: number of social contacts (friends) among representatives of their own ethnicity and among the Russian-speaking population, type of acculturation strategy, degree of life satisfaction, cultural and economic safety, and anxiety level.

## 1. Introduction

Last years’ social, economic and political changes have resulted in growth of intensive and extensive migration activity. The occurrence of compelled and voluntary migrants has brought the appearance and development of the new direction of social psychology, the psychology of migration—the circle of scientific psychological interests connected with the study of migration and immigration [1], adaptation [1,2], problems of migrants [3], assimilation and acculturation models [4,5], and processes of migration [6,7,8,9], mental health are studied [8,9], emotional well-being [10,11,12,13]. In the social-psychological literature, the various models of adaptation to the new conditions of the social-cultural environment are considered [14,15]. The various social-psychological features of migrants and emigrants such as ethnic identity [15,16], characteristics of psychological adaptation [16,17,18,19]. In the Russian scientific literature, the following problems are considered: the emigrants’ experience of interaction with the representatives of foreign culture [1,2,3,4], the features of ethnic identity [5,6,7,8,9,10,11], the degree of cultures similarity [11,12], the personal features of the emigrants [13,14,15], values of migrants [16,17,18,19] and many others. In Russia, the research of the influence of the social-cultural environment on processes of adaptation have started only in the last 10 to 15 years.

**Figure 1:**
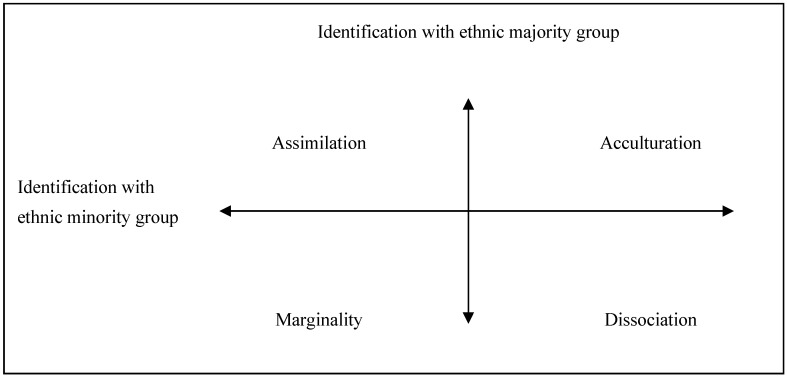
Model of identity of ethnic minority groups by N. Hutnik (1991) [8].

The new socio-psychological approach of understanding the process of adaptations of ethnic minority groups with the majority group was suggested by N. Hutnik [8,9]. According to Hutnik’s model, the ethnic identity of the minority comprises two main elements: the strategy of self-categorization and the cultural adaptation style—beliefs, relations, values, and ways of behaving. Both components are relevant to the ethnic minority group as well as to the ethnic majority group. There are four styles of cultural adaptation and four corresponding strategies of self-categorization (Figure 1).

In the four-polar model, created and approbated with a sample of Indian girls living in England, N. Hutnik suggested four strategies for ethnic self-identification of individuals: the assimilative strategy (an individual sees him/herself as belonging exclusively to the majority group), the acculturative strategy (an individual identifies him/herself with both the ethnic minority group and the majority group), marginality and dissociation [8].

**Figure 2:**
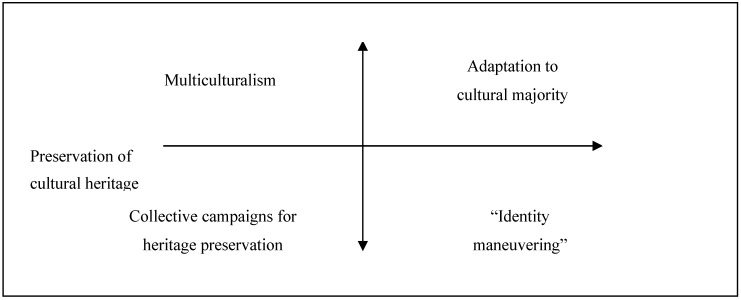
Model of integration strategies by F. M. Moghaddam [12].

The unique model of acculturation that is based on the social theory of identity is a mobility model of cultural integration, also known as a model of integration strategies, developed by F. Moghaddam. This model studies strategies used by immigrants to improve their economic and social status in Canada by studying their position in two dimensions: assimilation against preservation of cultural heritage, and normative behavior against non-normative behavior. The model provides four strategies of mobility [12] (Figure 2).

The author distinguishes the three main spheres in life of the migrants: private life, life within their ethnic society where norms and values of their “native” culture hold a dominant position, and public life where “new” values and norms prevail. Conception of normative and non-normative integration strategies means degree of correspondence between strategies and interethnic correlation of forces, set for the present moment. The strategies are referred to as normative if they do not break the status quo [12].

**Figure 3:**
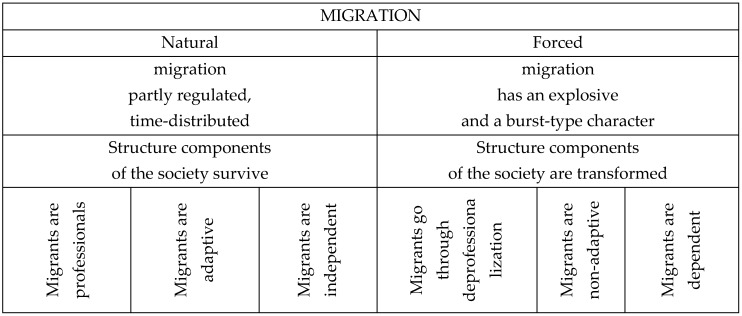
Natural and forced of migration [7].

The article contains a review of social, psychological, and demographic aspects of acculturation of migrants from three countries in Central Asia (the former Soviet republic)—Kyrgyzstan, Tajikistan, and Uzbekistan—living in Russia (St. Petersburg). The research found both general and specific features related to certain ethnic groups. Migration can take two forms: forced or voluntary. The most difficult for regulation are forced forms of migrations, as they have a spontaneous and massive character, and transform the existing structure of the society. In general, the process of voluntary (natural) migration is a more regulated process. Structural components of the society do not experience any transformations due to voluntary migration (Figure 3).

Therefore, this research aimed to reveal social and psychological peculiarities of acculturation processes among migrants from the countries of Central Asia (Kyrgyzstan, Tajikistan, and Uzbekistan) in new social and cultural conditions (St. Petersburg, Russia), and to determine outer and inner factors that affect success in the acculturation process.

## 2. Object and Methods

The basic hypothesis of the research is as follows: there are both general and specific peculiarities of the acculturation process of migrant workers. There are significant differences in adaptation of the migrants from different countries of Central Asia.

### 2.1. Object of the Research

We selected labor migrants from Tajikistan, Kyrgyzstan, and Uzbekistan as respondents, considering data by the Federal State Statistics Service of Russia, which demonstrate that the majority of migrants’ inflow to Russia are labor migrants from these countries of Central Asia. All the respondents had legal permission to stay in the Russian Federation with the purpose of employment. Most of them stayed in the RF without families or children. Almost none of them had any private dwelling. Their level of knowledge of the Russian language varied from “know” and “understand” levels to fluent speaking (sometimes without any accent). The respondents were descendants from three countries with their own history, culture, and language. The survey was conducted in common conditions for all the respondents—at their workplaces or during leisure time.

### 2.2. Methods

The research was conducted in St. Petersburg. In total, 98 people aged from 19 to 42 years old took part in the research (median age= 32.26, SD = 3.44), among them: women—44%, men—56%. Three ethnic groups were represented in the selection: Kyrgyz people (34 persons), Tajik people (32 persons), and Uzbek people (32 persons). Distribution of respondents by basic social and demographic factors—sex, level of religious commitment, professional status, economic and marital status—in all the three reviewed ethnic groups was equal (we used chi-square, *p* > 0.05). The unique difference in distribution of the selection by demographic characteristics was their level of the education. For example, in the Tajik group, there were more specialists with secondary professional education than in the two other groups (*p* = 0.001) (Table 1).

The unique difference in distribution of the selection by demographic characteristics was level of education. Part of the Kyrgyz and the Uzbek groups had an academic degree or an uncompleted academic degree, and Kyrgyz people were more likely to be people with incomplete higher education (44.4%). Getting a higher education is honorable and prestigious for many migrants and their families, which is why many parents begin to save money on the education of their children from an early age. In Russia, this is one of the main reasons for being separated from one’s children (Table 2).

For example, in the Tajik group, there were more specialists with the secondary professional education than in two other groups (*p* = 0.001); more than a half of the Kyrgyz and the Uzbek groups had an academic degree or an uncompleted academic degree. The method used for research was the comprehensive study of acculturation developed by J. Berry for the international project MIRIPS (Mutual Intercultural Attitudes in Plural Societies—the MIRIPS Projective) [2]. The procedure includes 27 questionnaires, including 23 for the migrants. The questionnaires for migrants are supposed to obtain the following information: personal details; level of civil and ethnic identity; evaluation of cultural, economic, and physical safety; acculturation settings; conceived discrimination; attitude to representatives of other ethnic groups (“thermometer”), self-satisfaction and life-satisfaction; evaluation of depression and uneasiness; and evaluation of social and cultural inadaptation. The method elicits strategies of acculturation used by the migrants in the process of interaction with the dominant population that consequently may be considered as a factor of successful adaptation.

The data obtained in the research were analyzed using mathematic and statistical processing in the SPSS 21.0 software. For statistical analysis, we used Student’s *t*-test to define credibility of different averages and multivariate regression analysis MANOVA (analysis of variance) to elicit correlation in factors. As independent variables in the research, we considered the following: scale of settings for acculturation strategies (separation, marginalization, assimilation, integration) and self-satisfaction and life-satisfaction. To analyze the results against each scale, calculating the average values of answers in the corresponding groups of questions was required. To reduce the possibility of using typical answers, we used reverse questions.

## 3. Results

### 3.1. Knowledge of the Russian Language

First, we should note that minimum values of knowledge of the Russian language demonstrated by the selection were between 2.5 and 3.8 points. This confirms that the respondents had sufficient knowledge of the Russian language. In most of the participants’ childhood, their countries were republics of the USSR. Russian was the state language and obligatory in all schools. In general, in the selection from the three ethnic groups, women evaluated their knowledge of the Russian language higher (*p* = 0.049), and spoke it more often (*p* = 0.045). The most significant difference between men and women was found in the Uzbek group. In the Kyrgyz group, the knowledge of the Russian language was the opposite, being higher for men, but the difference was insignificant. The most significant difference between men and women was found in the Uzbek group. In the Kyrgyz group, the knowledge of the Russian language was also the opposite, being higher for men, but the difference was also in significant. The fact that women know the Russian language better than men may be partially explained by the specificity of their work, which usually involves social interaction with the local population (shops, public utilities, social institutions), whereas male migrants were mostly involved in physical labor and often worked in groups of their countrymen (such as in brigades at construction sites).

### 3.2. Social Environment (Friends)

In answers to the question about the nationality of their close friends, we found the significant difference between men and women in all the groups. Women more often claimed having friends among their own ethnic group and Russian people (*p* = 0.006) and Russians (*p* = 0.024). At the same time, Tajik and Uzbek people meet with their friends significantly more often than Kyrgyz people. This is equally true for their friends of the same ethnicity (*p* = 0.015 and *p* = 0.004, respectively) and for their Russian friends (*p* = 0.000 and *p* = 0.000, respectively). Thus, women have more social contact with local people than men. At the same time, the Kyrgyz respondents significantly less often meet with their friends compared to the Tajik and Uzbek people.

### 3.3. Safety Issues

**Figure 4:**
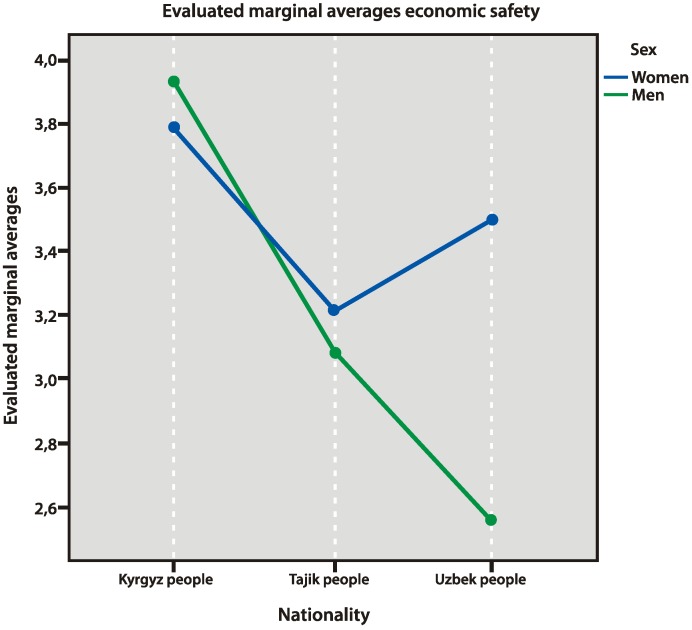
Economic safety factors.

Three types of safety have been studied in this work: economical, physical, and cultural. Kyrgyz people felt more economically safe, especially compared to labor migrants from Uzbekistan (*p* = 0.032). Uzbek men felt the least economically safe (Figure 4).

**Figure 5:**
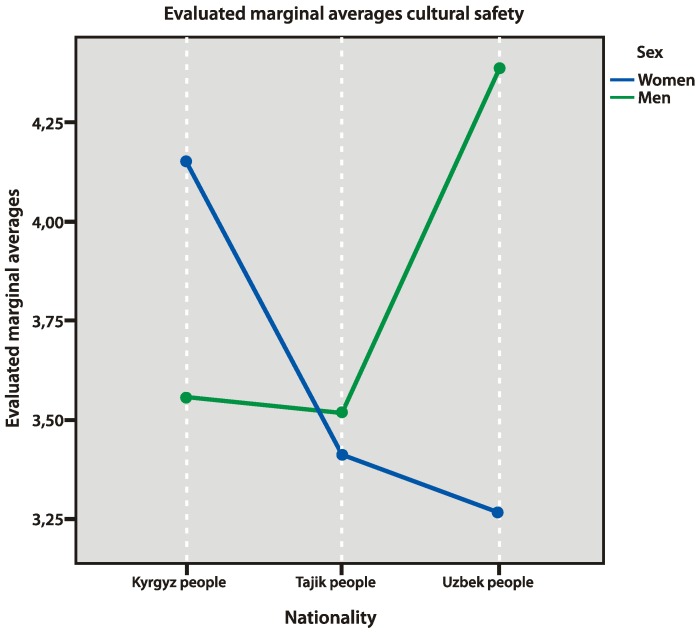
Cultural safety factors.

**Figure 6:**
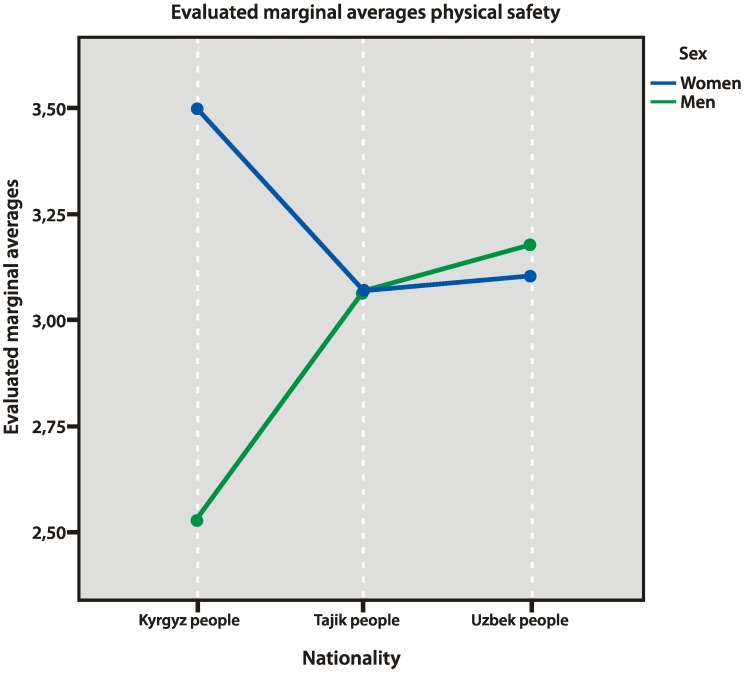
Physical safety factors.

The feeling of cultural safety refers to the desire for preservation and protection of one’s national traditions, values, lifestyle, religion, and other factors. The research found that high values of this factor belonged to Uzbek men, who have “minor concern about the loss of the cultural identity” and, in staying in Russia, “feel that their culture is safe”. On the other hand, Uzbek women were more concerned with losing their cultural identity and had a stronger tendency to protect their own culture from external influences (*p* = 0.005). On the contrary, in the group of Kyrgyz people, women felt more culturally safe than men (Figure 5 and Figure 6).

Kyrgyz men felt the least physically safe among all the categories of respondents, whereas Kyrgyz women felt the safest in the physical aspect (*p* = 0.032), believing that, in modern conditions, “chances to live a safer and easier life are higher than ever before” (Figure 6).

It is possible that the sense of physical insecurity in Kyrgyz men was due to their having less friends compared to Kyrgyz women and representatives of other ethnic groups. Availability of a wide social circle may serve as a kind of buffer, helping to comprehend the ambient environment as less dangerous and unpredictable.

### 3.4. Strategies of Acculturation

**Figure 7:**
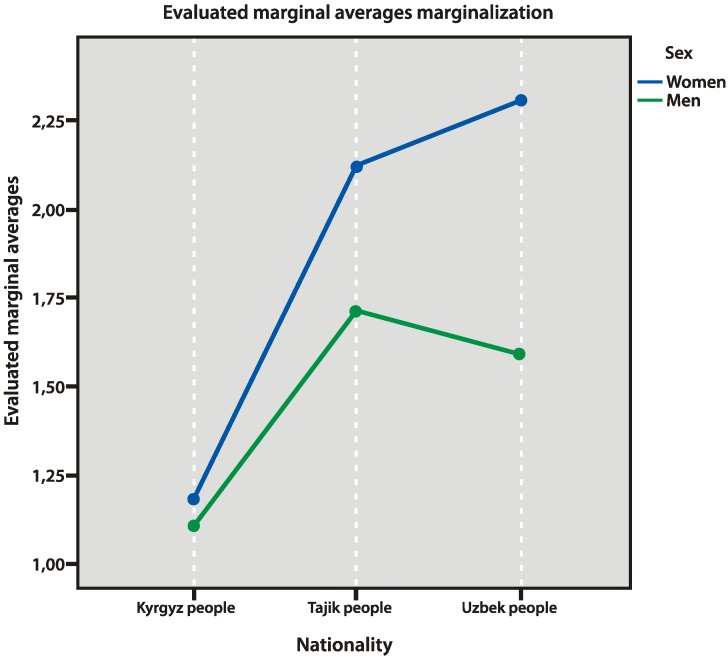
Strategy of marginalization.

**Figure 8:**
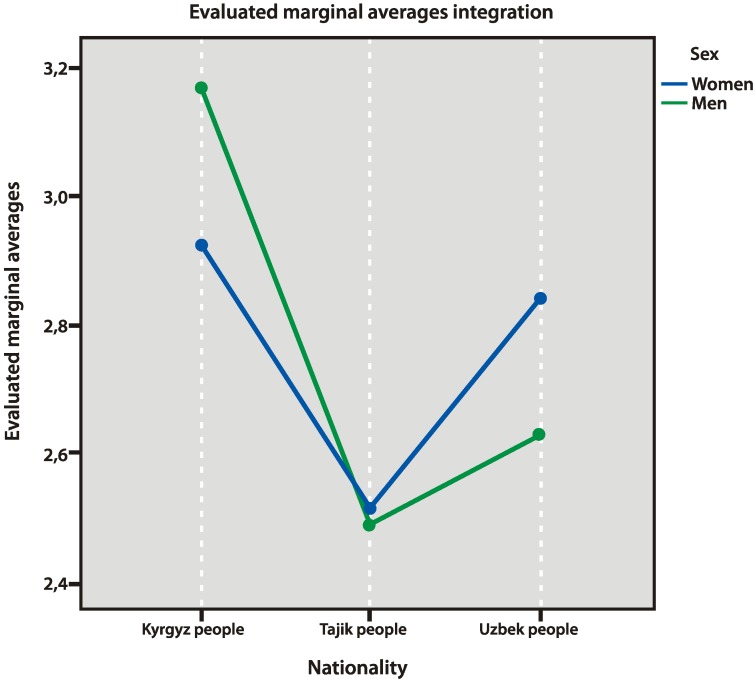
Strategy of integration.

When observing acculturation strategies of the respondents, we elicited significant differences in strategies of marginalization and integration (Figure 7 and Figure 8). Uzbek and Tajik people used the strategy of marginalization more often than Kyrgyz people (*p* = 0.000 and *p* = 0.000, respectively). Kyrgyz people significantly more often used the strategy of integration (compared to Tajik people, *p* = 0.000). At the same time, women from Uzbekistan significantly more often used the strategy of marginalization (*p* = 0.000). The latter data may be explained by their low evaluations of cultural safety.

### 3.5. Self-Satisfaction

**Figure 9:**
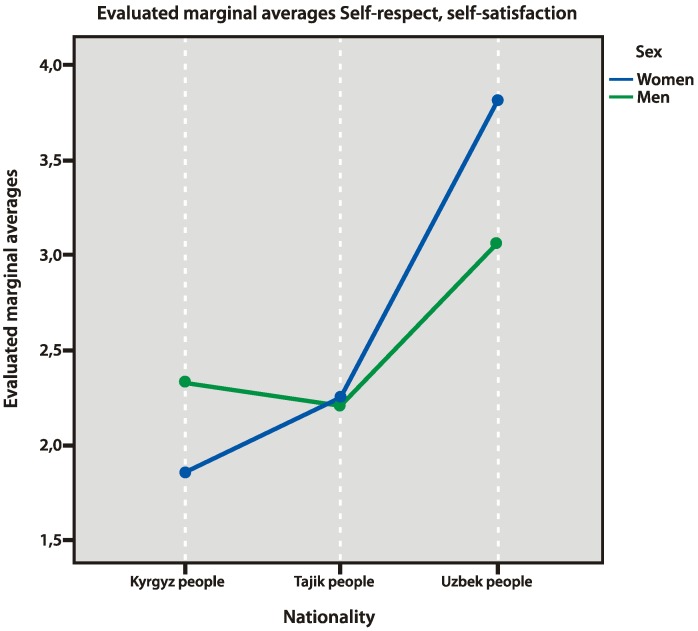
Self-satisfaction factors.

For self-satisfaction of the respondents, we elicited gender and ethnic peculiarities. Generally, women in the selection were found to be more self-satisfied than men (*p* = 0.004). This may be especially true for Uzbek women (*p* = 0.001), who were “generally self-satisfied” and “feel useful”. At the same time, Kyrgyz people demonstrated the lowest values of self-satisfaction compared to Uzbek people. They wanted “to be respected more” and believed that they were “worth respecting just like others” (*p* = 0.003) (Figure 9).

It should be noted that there were some contradictions in the results obtained from the selection of Uzbek women: low evaluation of perceived cultural safety, high degree of marginalization, whereas their self-satisfaction was higher than that of the other participants. In the group of Uzbek women, we found the highest false scale of factors, which may be explained by the significant problems with adaptation and by their reluctance to unveil them. Low self-satisfaction of Kyrgyz people may be explained by their elicited fears concerning their physical safety, as well as their narrow social circuit (friends) that might ease those fears. It should be noted that they worked in more qualified jobs than the other respondents, and they more often worked with Russian people than with their own compatriots. Moreover, Kyrgyz respondents (men and women) demonstrated the highest level of anxiety, especially compared to the people from Tajikistan (*p* = 0.003).

### 3.6. False Scale

**Figure 10:**
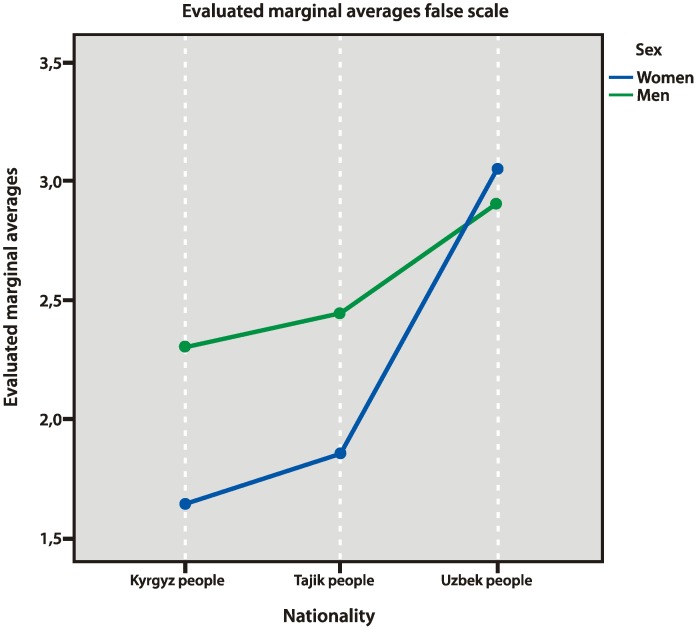
False scale factors.

The results obtained with the false scale (settings for socially advisable answers) demonstrated the gender and ethnic peculiarities. Generally, the male selection gave more socially approved answers (*p* = 0.000), whereas the maximum number of the advisable answers were given by the Uzbek selection (especially women), compared to the representatives of Kyrgyzstan (*p*= 0.000) and Tajikistan (*p* = 0.008; Figure 10).

## 4. Discussion

The configuration of factors by the indicators of adaptation of migrant workers from Kyrgyzstan, Tajikistan, and Uzbekistan studied in the research included both common features and specific differences. Thus, in general, the selection demonstrated a sufficiently high level of knowledge of the Russian language, which is a predictor for intercultural adaptation. Educational level of the respondents was no less than secondary or professional (52%), one third of them having an uncompleted academic degree and about 20% having a completed academic degree, which may also enhance successful adaptation to another culture. On average, women demonstrated better knowledge of the Russian language; they had a wider circle of contacts among both their compatriots and the local population. They also appeared more sincere than men, who more often gave socially approved answers.

The research results showed that there were significant differences between migrants from Kyrgyzstan, Tajikistan, and Uzbekistan by the following indicators of acculturation: number of social contacts (friends) among representatives of their own ethnicity and the Russian-speaking population; type of acculturation strategy; degree of life satisfaction; cultural, physical, and economic safety; anxiety level.

Representatives of Kyrgyzstan had a relatively high educational level (61% had a degree of higher education or unfinished higher education), performed more qualified work, more often followed the strategy of integration, had stronger feeling of economic safety, and were more honest in their answers. At the same time, the Kyrgyz respondents demonstrated low self-satisfaction and high degree of anxiety. Insufficient participation in social contacts (compared to representatives of other ethnic groups) may enhance feelings of uncertainty and anxiety, where even sufficient economic safety does not contribute to self-satisfaction and life-satisfaction.

The respondents from Tajikistan had a lower level of education (72.2% had secondary and secondary professional education); they involved in unqualified work more often than representatives of other groups; they tended to follow the marginalization strategy; and demonstrated low levels of economic, physical, and cultural safety. Both men and women had relatively high numbers of friends of different nationalities. Their levels of self-satisfaction were low. The members of this group had a low level of aspirations, whereas their anxiety level was the lowest in the selection.

The results obtained for the Uzbekistan group seemed controversial. About 60% of its members had a degree of higher education and unfinished higher education, whereas the same proportion was involved in unqualified work. They had a large number of social contacts, with both their compatriots and representatives of other ethnic groups. At the same time, they more often used the strategy of marginalization, which implies rejection of native cultural traditions and non-acceptance of traditions of the ethnic majority. However, Uzbek men were found to feel less secure in terms of economic factors, and at the same time they scored as having significantly high values of cultural safety. The respondents did not feel physically or economically secure—anxiety factors were high. Nevertheless, they evaluated their self-satisfaction higher than in any other group. High values on the self-satisfaction scale in combination with relatively high values on the anxiety scale may be explained by the lack of frankness, which may be confirmed by high values on the false scale. Presumably, an unfavorable psychological situation forced people to hide their actual condition by “demonstrating” socially favorable reactions.

Our respondents during the answering of the tests showed classical psychological phenomena, such as effect of ethnic facilitation, ethnic inhabitation, group synergism, group identity, group favoritism, and others. Most of them are presented and described in Table 2.

## 5. Conclusions

According to the results of the research performed, we should admit that the labor migrants from Central Asia had a high potential for adaptation to the Russian culture, manifested primarily in their knowledge of the Russian language, which contributes to the development of social contacts and adaptation of new behavioral patterns. The educational level of the migrants enables them to get involved in more qualified work, thus increasing their self-respect and self- and life-satisfaction. Within this study, we tried to perform a detailed analysis of the social and psychological characteristics of the labor migrants—settings and strategies of acculturation, ethnic identity, and success of adaptation. The main results of this work were elicitation and detailed description of the psychological effects in each of the groups under consideration, and in general and specific patterns of acculturation in each group of the labor migrants. All the above confirmed the necessity of reviewing the migration in dynamics, considering the cultural, social, and psychological peculiarities of migrants.

Indeed, the limitations of this work include the lack of possibility to perform longitudinal research. However, its key results have formed the basis for further research at Saint Petersburg State University. Another limitation concerns the relatively low number of participants in the three groups of respondents. This limitation was neutralized by the mathematical methods used for data processing.

The obtained results will make it possible to modify the existing programs aimed at controlling the processes of migration and acculturation of labor migrants from the countries of Central Asia. However, the need for further research is high, as labor migrants, initially coming for short periods (to earn money), afterwards (under the influence of certain factors) change their settings and prefer not to return or postpone their return to their native country. This may lead to antagonism and a reaction of the host community towards labor migrants.

On the other hand, migration inflows bring in other cultures, values, and traditions, which may not be “better or worse”, but come in contact with the host culture, resulting in an acculturation process. Therefore, the host society may reevaluate and appreciate the values of their own culture at a new level.

It is evident that the host side (host society) bears a certain degree of responsibility for whom it hosts, for what period, and on which terms. It is necessary to study the settings of the host society in relation to different types of migrants, to elicit its degree of tolerance, as well as causes of dislike, fears, and concerns.

In conclusion, it should be stated that people must not be deprived of their right to search for better conditions of life. However, social psychologists, among others, are responsible for making this process reasonable, manageable, and controllable.

## Figures and Tables

**Table 1:** Professional level of migrants.

			Nationality			
			Kyrgyz	Tajik	Uzbek	Total
Professional level	Unskilled workers	% nationality	38.9%	72.2%	58.3%	56.3%
	Skilled workers	% nationality	27.8%	27.8%	41.7%	31.3%
	Managers	% nationality	33.3%	0.0%	0.0%	12.5%
Total			100%	100%	100%	100%

**Table 2:** The level of migrants.

			Nationality			
			Kyrgyz	Tajik	Uzbek	Total
Educational level	Secondary special education	% nationality	38.9%	72.2%	41.7%	52.1%
	Incomplete higher	% nationality	44.4%	16.7%	33.3%	31.3%
	Higher education	% nationality	16.7%	11.1%	25.0%	16.7%
Total			100%	100%	100%	100%
